# Supplementary material for: Causal Relationship between Aging and Anorexia Nervosa: A White-Matter-Microstructure-Mediated Mendelian Randomization Analysis
Source: Biomedicines. 2024 Aug 16;12(8):1874. doi: 10.3390/biomedicines12081874 (PMC11351342; doi:10.3390/biomedicines12081874)
Supplement: Supplementary file 1 [file biomedicines-12-01874-s001.zip › Supplementary Figure/Figure S7/Single SNP plot of MR analysis for ubm-b-1777 to AN.pdf]

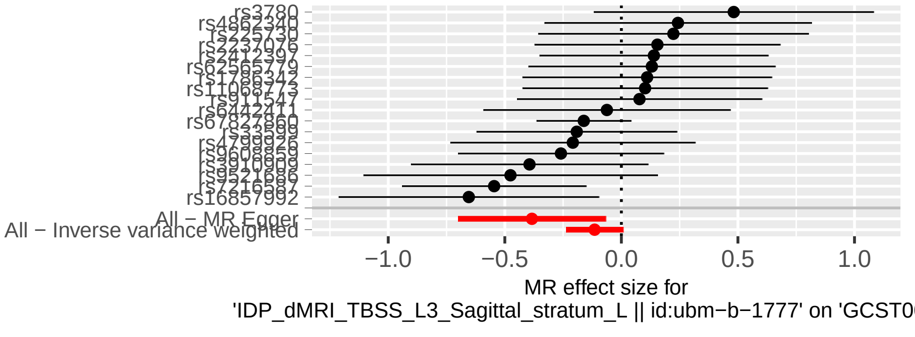

rs3780
rs4862340
rs225730
rs2237076
rs2412397
rs62565779
rs1786342
rs11068773
rs911547
rs6442411
rs67827860
rs33599
rs4799926
rs9606859
rs3910909
rs9521686
rs7216587
rs16857992
All − MR Egger
All − Inverse variance weighted
−1.0
−0.5
0.0
0.5
1.0
MR effect size for
'IDP_dMRI_TBSS_L3_Sagittal_stratum_L || id:ubm−b−1777' on 'GCST0
